# Supplementary material for: PD-L1-directed PlGF/VEGF blockade synergizes with chemotherapy by targeting CD141+ cancer-associated fibroblasts in pancreatic cancer
Source: Nat Commun. 2022 Oct 22;13:6292. doi: 10.1038/s41467-022-33991-6 (PMC9588060; doi:10.1038/s41467-022-33991-6)
Supplement: Supplementary file 1 — Supplementary Information [file 41467_2022_33991_MOESM1_ESM.pdf]

**Supplementary Information**

Materials and Methods, Table, Supplementary Figures 1–9 and Legends

# PD-L1-directed PlGF/VEGF blockade synergizes with chemotherapy by targeting CD141$^+$ cancer-associated fibroblasts in pancreatic cancer

Duk Ki Kim[1,2,3,4†], Juhee Jeong[3†], Dong Sun Lee[2], Do Young Hyeon[5], Geon Woo Park[3], Suwan Jeon[3], Kyung Bun Lee[6], Jin-Young Jang[7], Daehee Hwang[5], Ho Min Kim[1,2]*, and Keehoon Jung[3,4]*

[1]Graduate School of Medical Science and Engineering, Korea Advanced Institute of Science and Technology (KAIST), Daejeon 34141, Republic of Korea

[2]Center for Biomolecular and Cellular Structure, Institute for Basic Science (IBS), Daejeon 34126, Republic of Korea

[3]Department of Anatomy and Cell Biology, Department of Biomedical Sciences, Seoul National University College of Medicine, Seoul 03080, Republic of Korea

[4]Institute of Allergy and Clinical Immunology, Seoul National University Medical Research Center, Seoul 03080, Republic of Korea

[5]School of Biological Sciences, Seoul National University, Seoul 08826, Republic of Korea

[6]Department of Pathology, Seoul National University College of Medicine, Seoul 03080, Republic of Korea

[7]Department of Surgery and Cancer Research Institute, Seoul National University College of Medicine, Seoul 03080, Republic of Korea

†These authors contributed equally to this work.

***Corresponding authors:** keehoon.jung@snu.ac.kr (K.J.), hm_kim@kaist.ac.kr (H.M.K.)

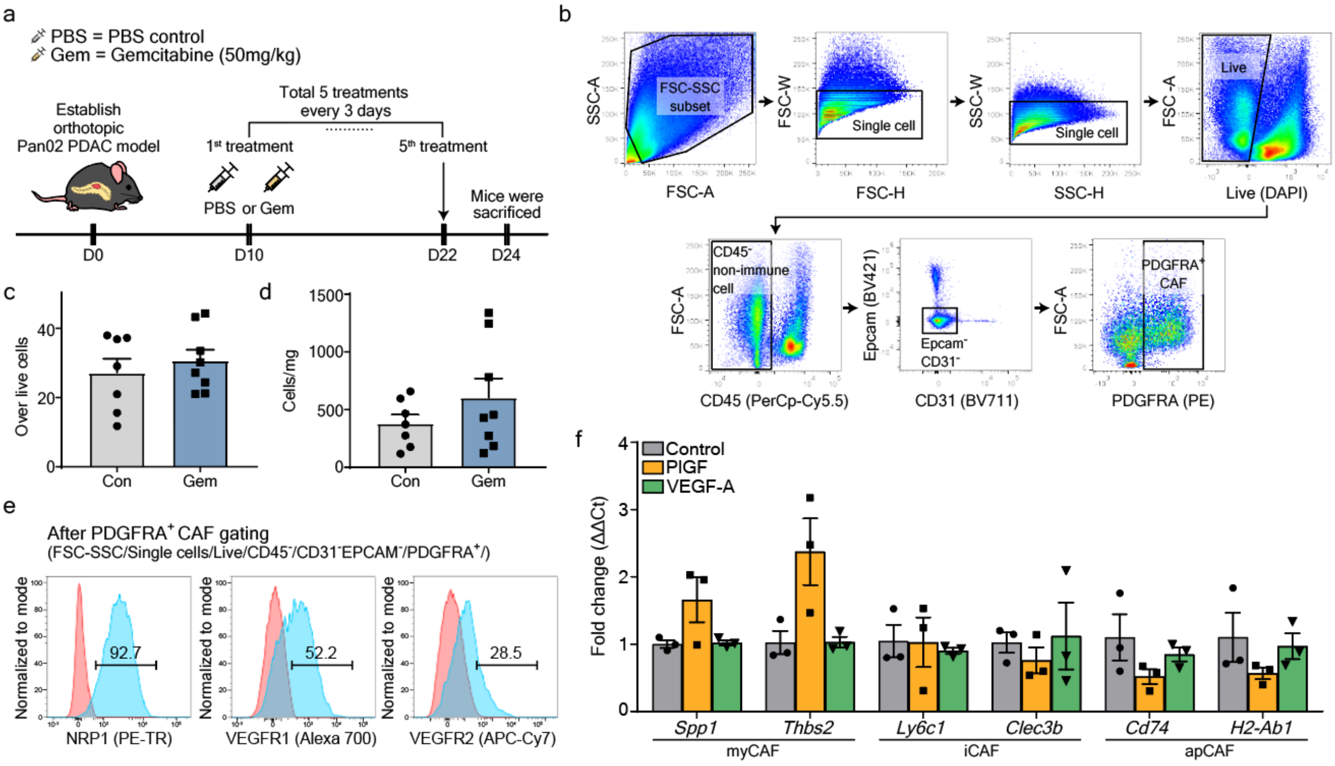


**Supplementary Figure 1 | Flow cytometry of CAFs in murine orthotopic Pan02 pancreatic tumor.**

(**a**) Experimental treatment scheme. Murine orthotopic pancreatic models were generated by implanting Pan02 cells ($5 \times 10^5$ cells/mouse) into the pancreas of 7–10-week old C57BL/6 mice. On day 10, tumor-bearing mice were intraperitoneally treated with either PBS (control) or gemcitabine (50 mg/kg, every 3 days). (**b**) Gating strategy for flow cytometry analysis of cancer-associated fibroblast (CAF) and its subsets in the tumor microenvironment (TME). CAFs were gated as FSC-SSC/Live/CD45$^-$/CD31$^-$EPCAM$^-$/PDGFRα$^+$ cells. Gating panel corresponds to FACS data in Supplementary Figure 1c-e, Supplementary Figure 1d, Figure 1g-h, Figure 3j, Figure 4f, Figure 5d and Supplementary Figure 5c. (**c, d**) Average (**c**) percentages and (**d**) absolute numbers of PDGFRα$^+$ CAFs in the TME were quantified via flow cytometry and compared between control (Con; n=7) and gemcitabine-treated (Gem; n=8) groups. (**e**) Representative flow cytometry plots showing the expression of VEGF-related receptors (NRP1, VEGFR1, and VEGFR2) in Pan02 tumor-infiltrating CAFs. (**f**) RT-PCR detection of myCAF (myofibroblastic CAF), iCAF (inflammatory CAF), and apCAF (antigen-presenting CAF) markers in response to PlGF or VEGFA. After serum starvation, PDGFRα$^+$ CAFs were treated with PlGF or VEGF for 6 h at 37°C. Data from three biological repeats were analyzed. Data are presented as

the mean ± SEM. two-tailed Student's t-test (c, d, f), vs. control. Source data are provided as a Source Data file.

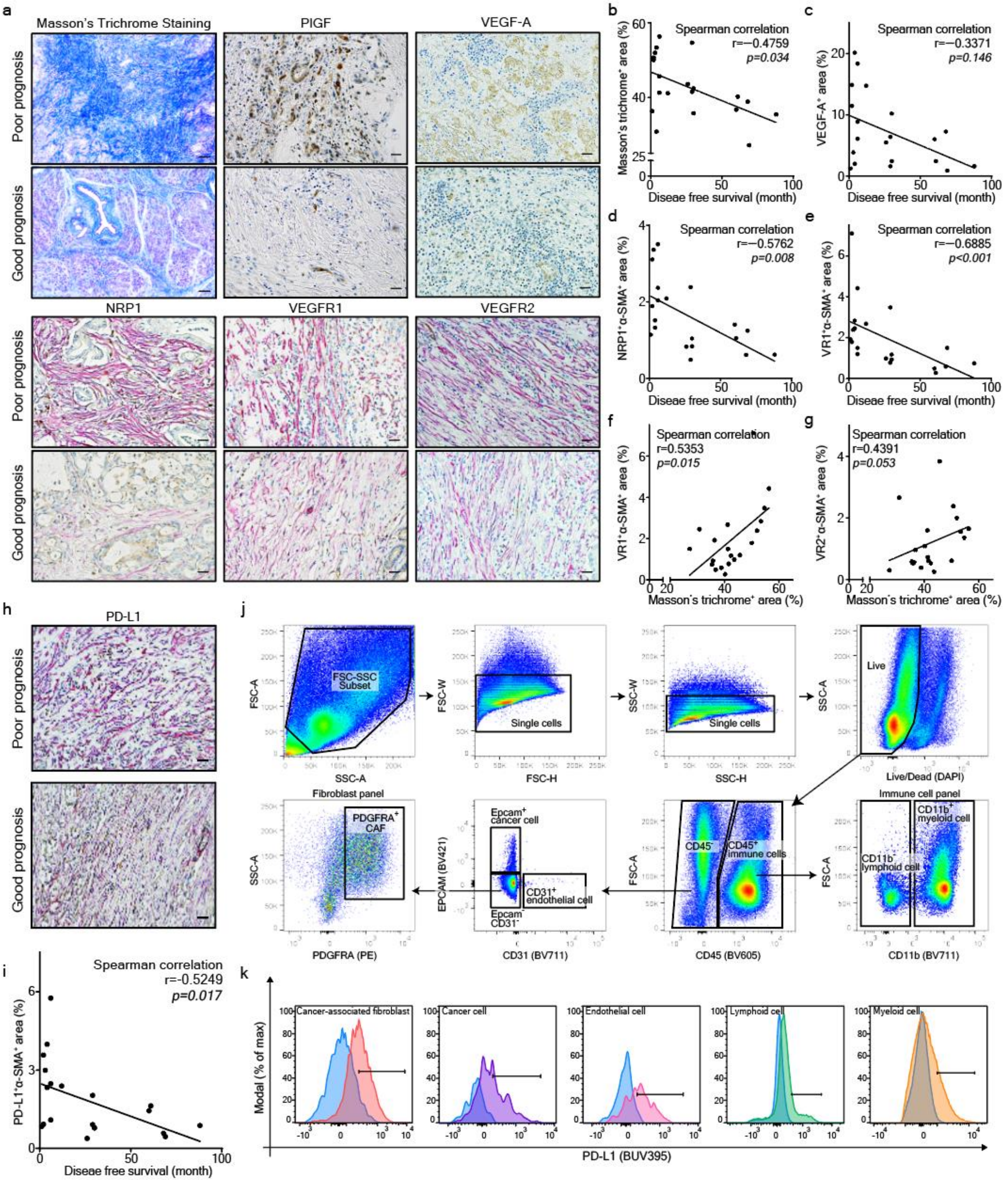


**Supplementary Figure 2 | Masson's trichrome staining and immunohistochemistry (IHC) staining of**

**patient-derived pancreatic adenocarcinoma tissues, and PD-L1 expressions in the Pan02 tumor microenvironment (TME).**

(**a**) Representative images of Masson's trichrome staining and IHC staining of patient-derived pancreatic tumors grouped by prognosis. PlGF, VEGFA, NRP1, VEGFR1 and VEGFR2 were stained with DAB, while $\alpha$-SMA was stained red. Scale bar, 100 $\mu$M. (**b-g**) Correlation analyses between disease-free survival of human PDAC patients and (**b**) tumor fibrosis (measured by Masson's trichrome$^+$ area%), (**c**) VEGFA$^+$ area%, (**d**) NRP1$^+\alpha$-SMA$^+$ area% or (**e**) VR1 (VEGFR1)$^+\alpha$-SMA$^+$ area%, and between tumor fibrosis and (**f**) VR1$^+\alpha$-SMA$^+$ area% or (**g**) VR2 (VEGFR2)$^+\alpha$-SMA$^+$ area%. Data from 20 PDAC patient tumor samples were analyzed (n=20). Statistical significance was accessed by two-tailed Student's t-test (b-g). **(h)** Representative IHC images of PD-L1$^+$ area (DAB color) and $\alpha$-SMA$^+$ area (red color) in human PDAC tumors grouped by patient prognosis. Scale bar, 100 $\mu$M. (**i**) Correlation analysis between disease-free survival of human PDAC patients and PD-L1$^+\alpha$-SMA$^+$ area%. Data from 20 PDAC patients' tumor samples were analyzed (n=20). (**j**) Flow cytometry gating strategy to identify indicated cellular populations. Gating panel corresponds to FACS data in Supplementary Figure 2k and Figure 2o. (**k**) Histogram showing PD-L1 expression on indicated populations (cancer-associated fibroblasts, cancer cells, endothelial cells, lymphoid cells, and myeloid cells) validated by flow cytometry. Histogram peaks are tinted with sky blue; FMO (Fluorescence minus one) control for PD-L1 (BUV395). Source data are provided as a Source Data file.

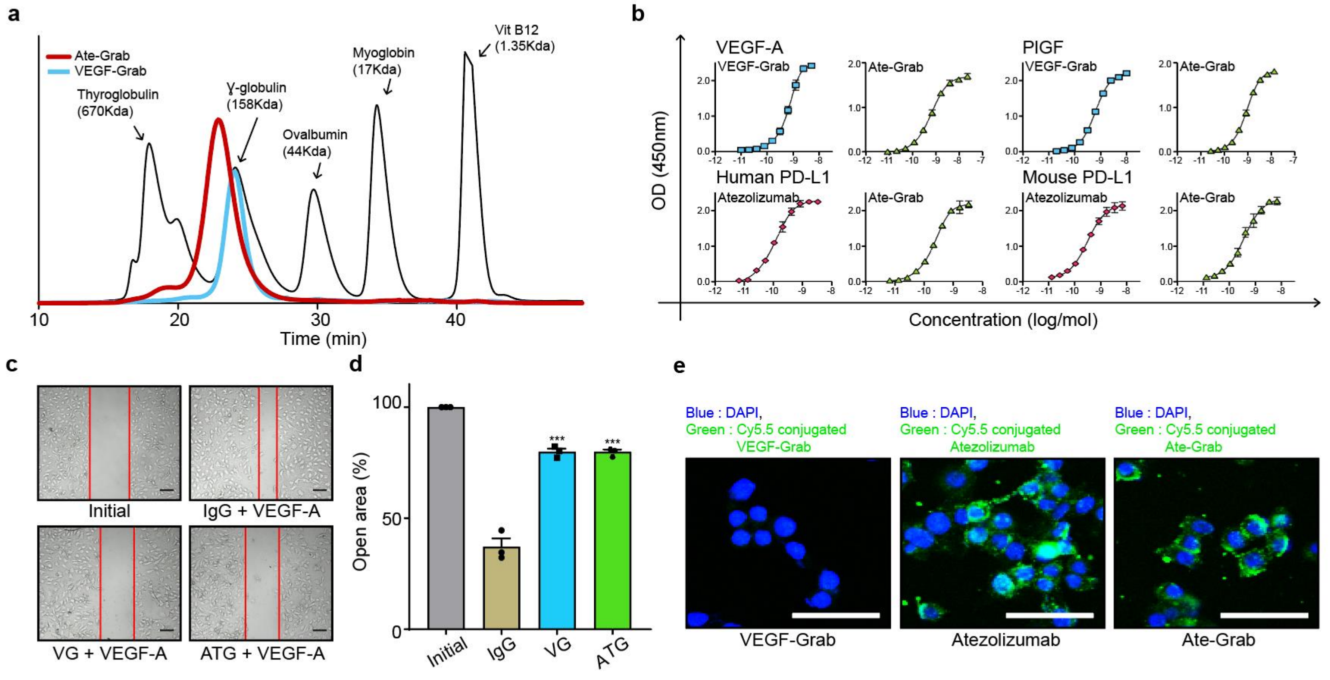


**Supplementary Figure 3 | Biochemical properties and biological effect of Ate-Grab at cellular level.**

(**a**) Size exclusion chromatography (SEC) of VEGF-Grab and Ate-Grab with gel filtration standard. Ate-Grab was produced in an Expi™ 297F expression system and purified via affinity chromatography. (**b**) Binding affinities of VEGF-Grab, Ate-Grab, and atezolizumab for target proteins (VEGF-A, PlGF, human PD-L1, and mouse PD-L1) were assessed via ELISA. n=3 independent samples were examined over 3 independent experiments. Data are presented as the mean ± SEM. (**c**) Representative images depict wound healing assay of HUVECs treated with each indicated protein combination (25 nM) in the presence of VEGF-A (1 nM). Wound-healing margins are indicated with red lines. Scale bar, 500 μm. (**d**) Migration areas were measured. Data from three biological repeats were analyzed. Data are presented as the mean ± SEM. ***P<0.001 versus IgG, One-way ANOVA with Tukey's multiple comparisons. (**e**) Confocal microscopy confirmed the surface binding of VEGF-Grab, atezolizumab, and Ate-Grab to PD-L1-expressing Pan02 cells. Blue color represents the nucleus of Pan02 cancer cells, and green color represents the drugs bound to PD-L1 on the surface of the cancer cells. The experiment was repeated for 3 times independently with similar results. Scale bars, 50 μm. Source data are provided as a Source Data file.

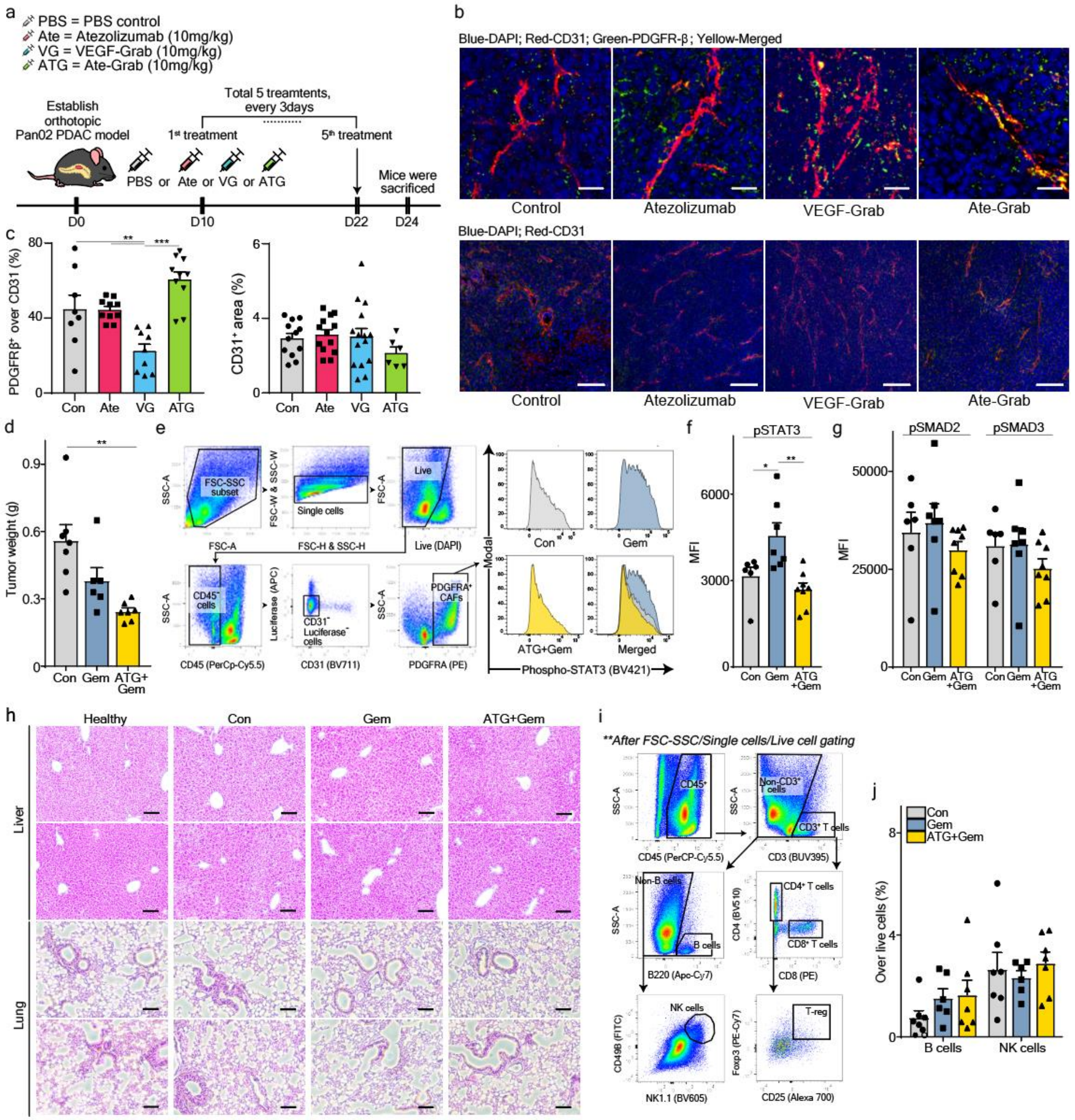


**Supplementary Figure 4 | Validation of the *in vivo* efficacy of Ate-Grab.**

(**a**) Experimental treatment scheme. When the tumor volume reached 50–100 mm$^3$, mice were intraperitoneally treated with either PBS (control; n=6) or atezolizumab (n=5), VEGF-Grab (n=4) and Ate-Grab (n=5). (**b-upper panel**) Representative immunofluorescence (IF) images staining for CD31 (red)

and PDGFR-β (green) in Pan02 tumors treated with different agents. Yellow indicates the co-expression of CD31 and PDGFR- β. Scale bar, 200 µm. (**b-lower panel**) Representative IF images staining for CD31 (red) in Pan02 tumors treated with different agents. Scale bar, 100 µm. (**c**) Relative quantifications of yellow regions ($CD31^+$ PDGFR-$\beta^+$; left; Con, n=8; Ate, n=10; VG, n=9; ATG, n=10) and red regions ($CD31^+$; right; Con, n=12; Ate, n=13; VG, n=15; ATG, n=6) in IF data were analyzed by ImageJ. **P<0.01 (Con vs VG: P=0.009, Ate vs VG: P=0.006), ***P<0.001, One-way ANOVA with Tukey's multiple comparisons. (**d**) Average tumor weights of each treatment group (Con, n=7; Gem, n=6; ATG+Gem, n=7). Data are presented as the mean ± SEM. **P<0.01 (P=0.001), One-way ANOVA with Tukey's multiple comparisons. (**e**) Gating strategy to evaluate PDGFR$\alpha^+$ CAF and representative plots of pSTAT3 expression in PDGFR$\alpha^+$ CAF. Gating panel corresponds to FACS data in Supplementary Figure 4f-g, Figure 7h and Figure 7i. (**f, g**) Average MFI (mean fluorescence intensity) values of (**f**) p-STAT3, (**g**) p-SMAD2 and p-SMAD3 compared across different treatment groups (Con, n=6; Gem, n=7; ATG+Gem, n=8). Data are presented as the mean ± SEM. *P<0.05 (Con vs Gem: P=0.031), **P<0.01 (Gem vs ATG+Gem: P=0.002), One-way ANOVA with Tukey's multiple comparisons. (**h**) Representative H&E images of liver and lung tissues. Scale bar, 100 µm. (**i**) Gating strategy to evaluate lymphoid subsets. Gating panel corresponds to FACS data in Supplementary Figure 4j, Supplementary Figure 5d-f, Figure 5e-j, Figure 7j, Figure 8k-l and Supplementary Figure 9f. The experiment was repeated for 3 times (n=3) independently with similar results. (**j**) Average percentages of B cells and NK cells in tumors from each treatment group (Con, n=7; Gem, n=6; ATG+Gem, n=7). Data are presented as the mean ± SEM. one-way ANOVA. Con, control; Gem, gemcitabine; ATG+Gem, Ate-Grab+gemcitabine. Source data are provided as a Source Data file.

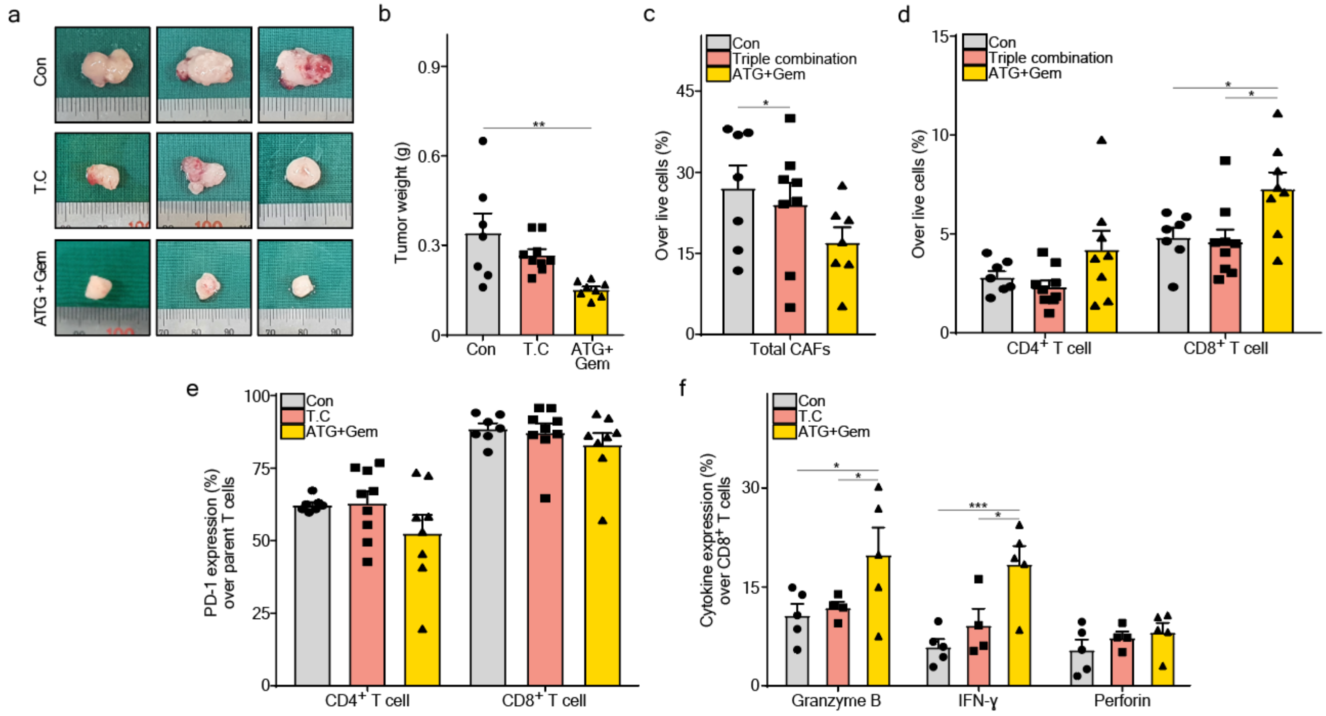


**Supplementary Figure 5 | Synergistic anti-tumor effect of Ate-Grab and gemcitabine over that of triple combination of atezolizumab, VEGF-Grab, and gemcitabine.**

(**a**) Representative images of Pan02 tumors from indicated treatment groups. Ruler scale, 1 mm. Con, control; T.C, triple combination (atezolizumab+VEGF-Grab+gemcitabine); ATG+Gem (Ate-Grab+gemcitabine). (**b**) Average tumor weights of each treatment group (Con, n=7; T.C, n=9; ATG+Gem, n=8). Data are presented as the mean ± SEM. **P<0.01 (P=0.004), One-way ANOVA with Tukey's multiple comparisons. (**c**) Average percentages of CAF in Pan02 tumor microenvironment measured by flow cytometry (Con, n=7; Gem, n=8; ATG+Gem, n=7). Data are presented as the mean ± SEM. *P<0.05; one-way ANOVA. (**d**) Average percentages of $CD4^+$ T cell and $CD8^+$ T cell in Pan02 tumor microenvironment, measured by flow cytometry (Con, n=7; Gem, n=9; ATG+Gem, n=8). Data are presented as the mean ± SEM. *P<0.05 (Con vs ATG+Gem: P=0.050, Triple combination vs ATG+Gem: P=0.021), One-way ANOVA with Tukey's multiple comparisons. (**e**) Average expression of PD-1 on $CD4^+$ T cell and $CD8^+$ T cell in Pan02 tumor microenvironment, measured by flow cytometry (Con, n=7; Gem, n=9; ATG+Gem, n=8). Data are presented as the mean ± SEM. one-way ANOVA. (**f**) Average expression of effector cytokines in Pan02 tumor-infiltrating CD8+ T cells, measured by flow cytometry (Con, n=5; Gem, n=4; ATG+Gem, n=5). Data are presented as the mean ± SEM. *P<0.05 (Granzyme B Con vs ATG+Gem: P=0.012, Granzyme B T.C vs ATG+Gem P=0.043, IFN-γ T.C vs ATG+Gem:

P=0.017), ***P<0.001, Two-way ANOVA with Tukey's multiple comparisons. Source data are provided as a Source Data file.

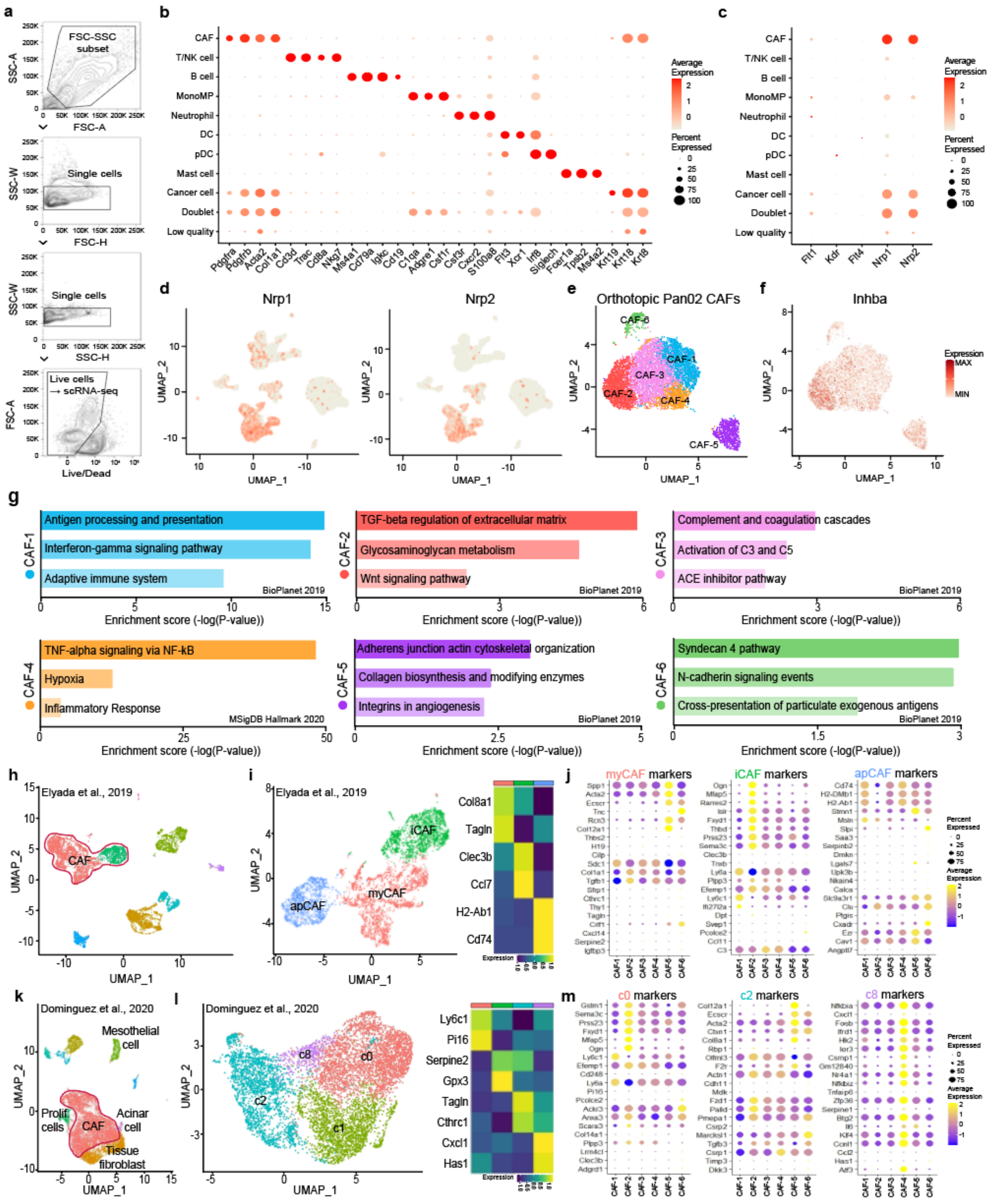


**Supplementary Figure 6 | Single-cell RNA sequencing of orthotopic Pan02 pancreatic tumors**

**treated with gemcitabine or a combination of Ate-Grab and gemcitabine.**

(**a**) Flow cytometry gating strategy for sorting tumor-infiltrating live cells. High cell viability should be ensured for successful single-cell RNA sequencing. (**b**) Dot plot showing the expression of multiple marker genes for precise cell type annotation prior to downstream analysis. (**c**) Dot plot and (**d**) feature plot showing VEGF-related receptor gene expression in Pan02 tumor-infiltrating cells. (**e**) Unbiased clustering of CAFs (cancer-associated fibroblasts) revealed six different CAF subpopulations (CAF-1, CAF-2, CAF-3, CAF-4, CAF-5, and CAF-6). (**f**) Feature plot showing *Inhba* expression in Pan02 CAFs. (**g**) Enrichment pathway analysis with the top 30 DEGs of each CAF subpopulation (*Enrichr*). P-value was delivered from two-sided Fisher's exact test. (**h**) Unbiased clustering (UMAP embedding) of KPC tumor scRNA-seq data from Elyada et al., 2019 (fibroblast enriched dataset) [1]. (**i**) Left: UMAP of zoom-in clustering of CAF sorted from (h). Right: Heatmap showing marker gene expression of myCAF, iCAF, and apCAF. (**j**) Dot plot showing the average expression of myCAF (myofibroblastic CAF; left), iCAF (inflammatory CAF; middle), and apCAF (antigen-presenting CAF; right) marker genes (Elyada et al., 2019) in orthotopic Pan02 CAF populations. (**k**) Unbiased clustering (UMAP embedding) of KPP tumor scRNA-seq data from Dominguez et al., 2020 [2]. (**l**) Left: UMAP of zoom-in clustering of CAF sorted from (k). Right: Heatmap showing marker gene expression of c0 CAF, c1 CAF, c2 CAF, and c8 CAF. (**m**) Dot plot showing the average expression of c0 CAF (left), myCAF-like c2CAF (middle), and c8 CAF (right) marker genes (Dominguez et al., 2020) in orthotopic Pan02 CAF populations. Source data are provided as a Source Data file.

a

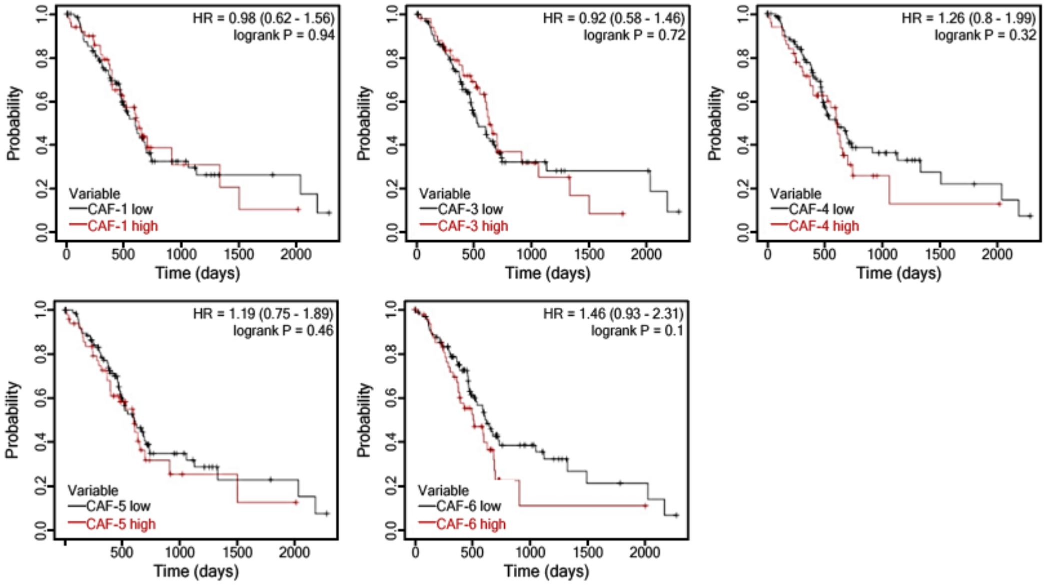
HR = 0.98 (0.62 - 1.56)
logrank P = 0.94
Variable
CAF-1 low
CAF-1 high
HR = 0.92 (0.58 - 1.46)
logrank P = 0.72
Variable
CAF-3 low
CAF-3 high
HR = 1.26 (0.8 - 1.99)
logrank P = 0.32
Variable
CAF-4 low
CAF-4 high
HR = 1.19 (0.75 - 1.89)
logrank P = 0.46
Variable
CAF-5 low
CAF-5 high
HR = 1.46 (0.93 - 2.31)
logrank P = 0.1
Variable
CAF-6 low
CAF-6 high
Probability
Time (days)

b

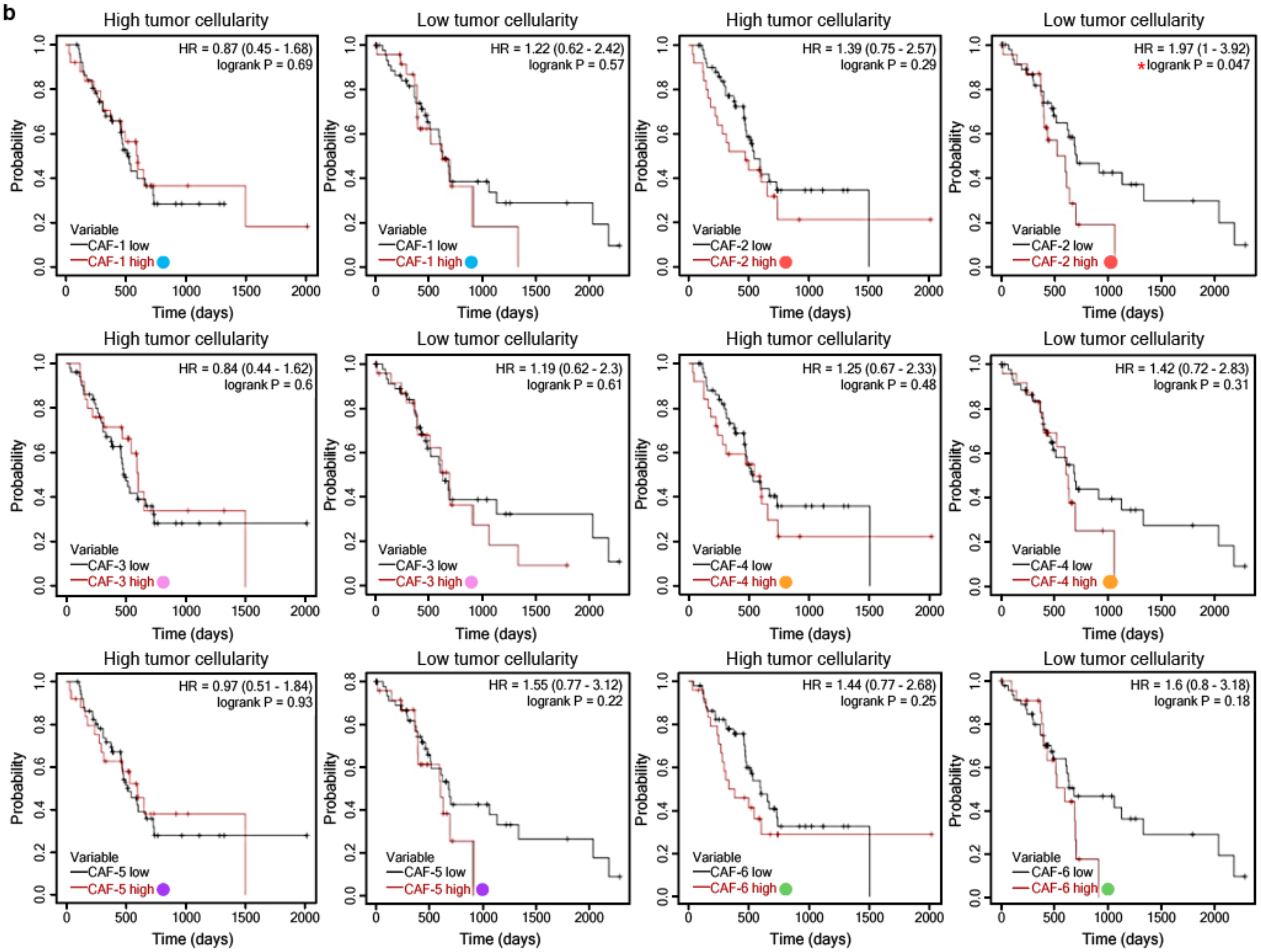
High tumor cellularity
HR = 0.87 (0.45 - 1.68)
logrank P = 0.69
Variable
CAF-1 low
CAF-1 high
Low tumor cellularity
HR = 1.22 (0.62 - 2.42)
logrank P = 0.57
Variable
CAF-1 low
CAF-1 high
High tumor cellularity
HR = 1.39 (0.75 - 2.57)
logrank P = 0.29
Variable
CAF-2 low
CAF-2 high
Low tumor cellularity
HR = 1.97 (1 - 3.92)
*logrank P = 0.047
Variable
CAF-2 low
CAF-2 high
High tumor cellularity
HR = 0.84 (0.44 - 1.62)
logrank P = 0.6
Variable
CAF-3 low
CAF-3 high
Low tumor cellularity
HR = 1.19 (0.62 - 2.3)
logrank P = 0.61
Variable
CAF-3 low
CAF-3 high
High tumor cellularity
HR = 1.25 (0.67 - 2.33)
logrank P = 0.48
Variable
CAF-4 low
CAF-4 high
Low tumor cellularity
HR = 1.42 (0.72 - 2.83)
logrank P = 0.31
Variable
CAF-4 low
CAF-4 high
High tumor cellularity
HR = 0.97 (0.51 - 1.84)
logrank P = 0.93
Variable
CAF-5 low
CAF-5 high
Low tumor cellularity
HR = 1.55 (0.77 - 3.12)
logrank P = 0.22
Variable
CAF-5 low
CAF-5 high
High tumor cellularity
HR = 1.44 (0.77 - 2.68)
logrank P = 0.25
Variable
CAF-6 low
CAF-6 high
Low tumor cellularity
HR = 1.6 (0.8 - 3.18)
logrank P = 0.18
Variable
CAF-6 low
CAF-6 high
Probability
Time (days)

**Supplementary Figure 7 | Kaplan-Meier overall survival curves of TCGA-PAAD patients.**

**(a)** Kaplan-Meier overall survival curves of 150 PAAD patients grouped based on the expression of the top 30 DEGs of CAF-1, CAF-3, CAF-4, CAF-5, and CAF-6 were analyzed respectively via the KM-plotter. HR, hazard ratio. CAF, Cancer-associated fibroblast. **(b)** Kaplan-Meier overall survival curves of 77 PAAD patients with high tumor cellularity (>= median value of 18.333) and 73 PAAD patients with low tumor cellularity (< median value of 18.333) grouped based on the expression of the top 30 DEGs of CAF-1~CAF-6 were analyzed respectively via the KM-plotter. HR, hazard ratio.

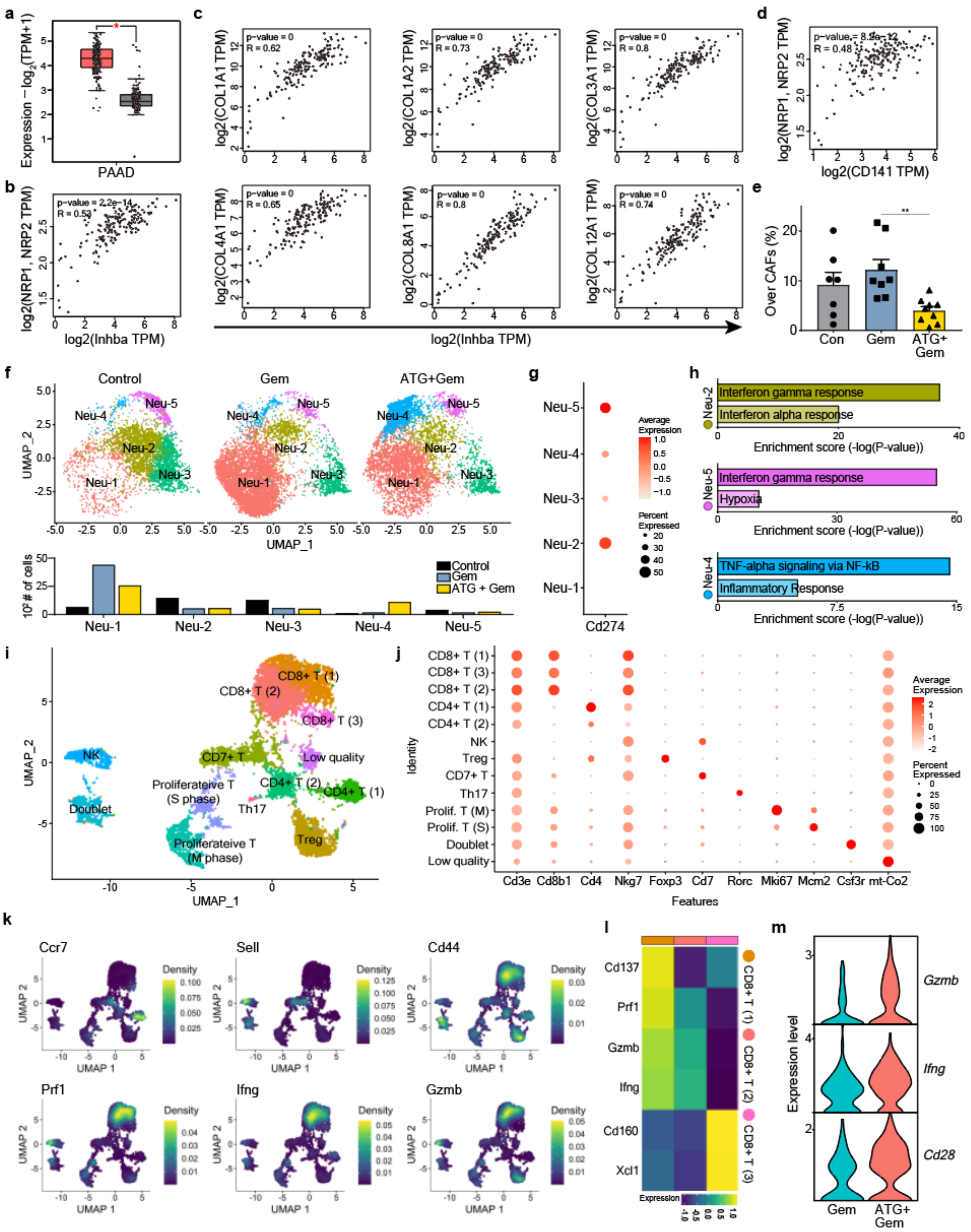

a
Expression − log2(TPM+1)
PAAD
b
log2(NRP1, NRP2 TPM)
log2(Inhba TPM)
p-value = 2.2e-14
R = 0.53
c
log2(COL1A1 TPM)
log2(COL1A2 TPM)
log2(COL3A1 TPM)
log2(COL4A1 TPM)
log2(COL8A1 TPM)
log2(COL12A1 TPM)
log2(Inhba TPM)
d
log2(NRP1, NRP2 TPM)
log2(CD141 TPM)
p-value = 8.9e-12
R = 0.48
e
Over CAFs (%)
Con
Gem
ATG+ Gem
f
Control
Gem
ATG+Gem
UMAP_1
UMAP_2
Neu-1
Neu-2
Neu-3
Neu-4
Neu-5
10^2 # of cells
Control
Gem
ATG + Gem
g
Cd274
Average Expression
Percent Expressed
h
Interferon gamma response
Interferon alpha response
Enrichment score (-log(P-value))
Hypoxia
TNF-alpha signaling via NF-kB
Inflammatory Response
i
CD8+ T (1)
CD8+ T (2)
CD8+ T (3)
NK
CD7+ T
Low quality
Proliferateive T (S phase)
CD4+ T (2)
CD4+ T (1)
Doublet
Th17
Proliferateive T (M phase)
Treg
j
Identity
CD4+ T (1)
CD4+ T (2)
Prolif. T (M)
Prolif. T (S)
Cd3e
Cd8b1
Cd4
Nkg7
Foxp3
Cd7
Rorc
Mki67
Mcm2
Csf3r
mt-Co2
Features
k
Ccr7
Sell
Cd44
Prf1
Ifng
Gzmb
Density
UMAP 1
UMAP 2
l
Cd137
Prf1
Gzmb
Ifng
Cd160
Xcl1
Expression
m
Expression level
Gzmb
Ifng
Cd28
Gem
ATG+ Gem

**Supplementary Figure 8 | Immunological changes induced by Ate-Grab in murine PDAC tumor microenvironment.**

(**a**) Comparison of expression patterns of top 40 DEGs (CAF-2) between human pancreatic cancer (TCGA-PAAD) and normal pancreatic tissue. Tumor (red), n=179; Normal (gray), n=171. *P<0.01 versus normal tissue. Statistical significance was accessed via two-tailed Student's t-test. Boxplot center represents median, bounds represent 25% and 75%, and whiskers show the minimum or maximum no further than 1.5 * interquartile range from the bound. (**b**) Pearson's correlation analysis between *Nrp* genes and *Inhba*. Data from 179 pancreatic cancer patients were analyzed. Statistical significance was accessed via two-tailed Student's t-test. (**c**) Pearson's correlation between multiple collagen genes and *Inhba*. Data from 179 pancreatic cancer patients were analyzed. Statistical significance was accessed via two-tailed Student's t-test. (**d**) Pearson's correlation analysis between *Nrp* genes and CD141. Data from 179 pancreatic cancer patients were analyzed. Statistical significance was accessed via two-tailed Student's t-test. (**e**) Flow cytometric analysis of the percentages of $CD141^{+}$ $MHC\text{-}II^{-}$ CAF-2 within total CAFs (Con, n=7; Gem, n=8; ATG+Gem, n=9). Data are presented as the mean ± SEM. **P<0.01 (P=0.009), Statical significance was accessed via one-way ANOVA with Tukey's multiple comparisons. Con, control; Gem, gemcitabine; ATG+Gem, Ate-Grab+gemcitabine. (**f**) Upper: Unbiased clustering of the integrated Pan02 tumor-infiltrating neutrophils (14,620 cells). Lower: Bar plot of the number of neutrophil subsets in untreated (Control), gemcitabine-treated (Gem), and Ate-Grab+gemcitabine-treated (ATG + Gem) Pan02 tumors. (**g**) Dot plot showing *Cd274* (PD-L1) expression in Pan02 tumor neutrophil subsets. (**h**) Enrichment pathway analysis with the top DEGs of each neutrophil subpopulation (*Enrichr*). P-value was delivered from two-sided Fisher's exact test. (**i**) Unbiased clustering of the integrated Pan02 tumor-infiltrating T/NK cells (7,622 cells). (**j**) Dot plot of the indicated features in each T/NK subpopulation. (**k**) Feature plots of the indicated mRNA features in the integrated T/NK subpopulations. (**l**) Heatmap of the indicated mRNA expression in three $CD8^{+}$ T subpopulations. (**m**) Violin plots of *Gzmb*, *Ifng*, and *Cd28* expression in gemcitabine and Ate-Grab+gemcitabine treatment groups. Source data are provided as a Source Data file.

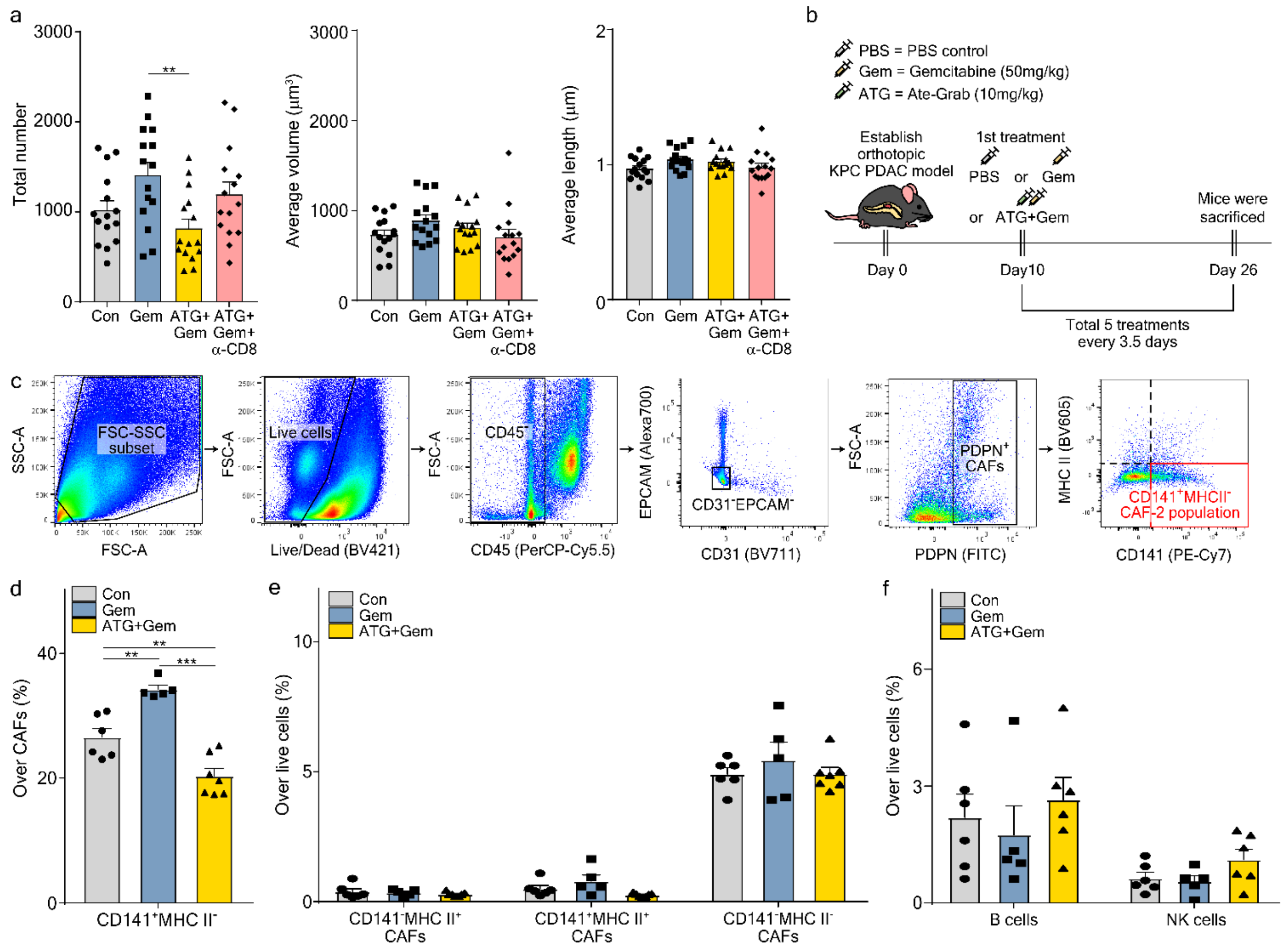


**Supplementary Figure 9 | CD8⁺ T cell-mediated therapeutic effect of Ate-Grab in both *KRAS*-mutated and *KRAS*-wild type murine PDAC.**

(**a**) Total number, average volume, and average length of 3D reconstructed collagen fibers in tumors compared across different treatment groups. Data from 15 randomly selected spots per group (three spots per one tumor x five tumors) were analyzed (n=15/group). Data are presented as the mean ± SEM. **P<0.01 (P=0.006); one-way ANOVA with Tukey's multiple comparisons. Con, control; Gem, gemcitabine; ATG+Gem, Ate-Grab+gemcitabine; ATG+Gem+α-CD8, Ate-Grab+gemcitabine+anti-CD8 antibody. (**b**) Experimental treatment scheme. Murine orthotopic pancreatic models were generated by implanting KPC001 cells ($5 \times 10^5$ cells/mouse) into the pancreas of 7–10-week-old C57BL/6 mice. When the tumor volume reached 50–100 $mm^3$, tumor-bearing mice were intraperitoneally treated with either PBS (control), gemcitabine (50 mg/kg, every 3.5 days), or gemcitabine with Ate-Grab (10 mg/kg, every 3.5 days). (**c**) Flow cytometry gating strategy to evaluate PDPN⁺ CAF (cancer-associated fibroblast) in

KPC tumor and CAF subsets defined by MHC-II and CD141 expression. Gating panel corresponds to FACS data in Supplementary Figure 9d-e and Figure i-j. (**d**) Average percentages of $CD141^{+}MHC\text{-}II^{-}$ cells in total CAFs were measured and compared across indicated treatment groups (Con, n=6; Gem, n=5; ATG+Gem, n=7). Data are presented as the mean ± SEM. **P<0.01 (Con vs Gem: P=0.002, Con vs ATG+Gem: P=0.005), ***P<0.001; one-way ANOVA with Tukey's multiple comparisons. (**e**) Average percentages of CAF subsets in total tumor-infiltrating live cells were measured and compared across indicated treatment groups (Con, n=6; Gem, n=5; ATG+Gem, n=7). Data are presented as the mean ± SEM; one-way ANOVA. (**f**) Average percentages of B cells and NK cells in total tumor-infiltrating live cells were measured and compared across indicated treatment groups (Con, n=6; Gem, n=5; ATG+Gem, n=6). Data are presented as the mean ± SEM; one-way ANOVA. Source data are provided as a Source Data file.

**Supplementary Table 1 | List of primer sequences used for qRT-PCR analysis in this study**

| Genes | Gene ID | Sequence |
|---|---|---|
| *Col1a1* | NM_007742 | Forward: CCTCAGGGTATTGCTGGACAAC |
| | | Reverse: CAGAAGGACCTTGTTTGCCAGG |
| *Acta2* | NM_007392 | Forward: TGCTGACAGAGGCACCACTGAA |
| | | Reverse: CAGTTGTACGTCCAGAGGCATAG |
| *Fn1* | NM_010233 | Forward: CCCTATCTCTGATACCGTTGTCC, |
| | | Reverse: TGCCGCAACTACTGTGATTCGG |
| *Spp1* | NM_009263 | Forward: GCTTGGCTTATGGACTGAGGTC |
| | | Reverse: CCTTAGACTCACCGCTCTTCATG |
| *Thbs2* | NM_011581 | Forward: GTATGGAGGGAAGGACTGTGTC |
| | | Reverse: ACTTGGCTCCAGGAAAACACGG |
| *Ly6c1* | NM_010741 | Forward: GCGCCTCTGATGGATTCTGCAT |
| | | Reverse: ATCCCTGATTGGCACACCAGCA |
| *Clec3b* | NM_011606 | Forward: CGAGGAACTCAAGAACAGGATGG |
| | | Reverse: GCCTCATGGAAGGTCTTCGGTT |
| *Cd74* | NM_001042605 | Forward: GCTGGATGAAGCAGTGGCTCTT |
| | | Reverse: GATGTGGCTGACTTCTTCCTGG |
| *H2-Ab1* | NM_207105 | Forward: GTGTGCAGACACAACTACGAGG |
| | | Reverse: CTGTCACTGAGCAGACCAGAGT |
| *Gapdh* | NM_008084 | Forward: CATCACTGCCACCCAGAAGACTG |
| | | Reverse: ATGCCAGTGAGCTTCCCGTTCAG |

*Col1a1*, collagen type I alpha 1; *Acta2*, actin alpha2, smooth muscle; *Fn1*, fibronectin 1; *Spp1*, secreted phosphoprotein 1; *Thbs2*, thrombospondin-2; *Ly6c1*, lymphocyte antigen 6 complex, locus C1; *Clec3b*, C-type lectin domain family 3 member B; *Cd74*, cluster of differentiation 74; *H2-Ab1*, histocompatibility 2, class II antigen A, beta ; *Gapdh*, glyceraldehyde-3-phosphate dehydrogenase.

**Supplementary Table 2 | List of clinical characteristics of patients in this study**

| Patient | Stage | Differentiation | Recurrence |
|---|---|---|---|
| PDAC011 | II | MD | Y |
| PDAC076 | II | PD | Y |
| PDAC085 | II | PD | Y |
| PDAC087 | II | PD | Y |
| PDAC180 | II | PD | Y |
| PDAC186 | II | unstated | Y |
| PDAC199 | II | MD | Y |
| PDAC234 | II | MD | Y |
| PDAC257 | II | unstated | Y |
| PDAC258 | II | MD | Y |
| PDAC037 | II | MD | N |
| PDAC132 | I | MD | Y |
| PDAC156 | II | WD | N |
| PDAC213 | II | MD | N |
| PDAC247 | I | MD | N |
| PDAC251 | I | WD | Y |
| PDAC253 | I | MD | N |
| PDAC254 | II | PD | Y |
| PDAC255 | II | WD | Y |
| PDAC269 | II | MD | Y |

WD, well differentiated; MD, moderately differentiated; PD, poorly differentiated; Y, yes; N, no.

**Supplementary References**


1 Elyada, E. et al. Cross-Species Single-Cell Analysis of Pancreatic Ductal Adenocarcinoma Reveals Antigen-Presenting Cancer-Associated Fibroblasts. *Cancer Discovery* **9**, 1102-1123, doi:10.1158/2159-8290.CD-19-0094 %J Cancer Discovery (2019).

2 Dominguez, C. X. et al. Single-Cell RNA Sequencing Reveals Stromal Evolution into LRRC15+ Myofibroblasts as a Determinant of Patient Response to Cancer Immunotherapy. *Cancer Discovery* **10**, 232-253, doi:10.1158/2159-8290.CD-19-0644 %J Cancer Discovery (2020).
