# Supplementary material for: PD-L1-directed PlGF/VEGF blockade synergizes with chemotherapy by targeting CD141+ cancer-associated fibroblasts in pancreatic cancer
Source: Nat Commun. 2022 Oct 22;13:6292. doi: 10.1038/s41467-022-33991-6 (PMC9588060; doi:10.1038/s41467-022-33991-6)
Supplement: Supplementary file 3 — Reporting Summary [file 41467_2022_33991_MOESM3_ESM.pdf]

nature portfolio

Corresponding author(s): Ho Min Kim, Keehoon Jung

Last updated by author(s): Sep 26, 2022

Reporting Summary

Nature Portfolio wishes to improve the reproducibility of the work that we publish. This form provides structure for consistency and transparency in reporting. For further information on Nature Portfolio policies, see our Editorial Policies and the Editorial Policy Checklist.

Statistics

For all statistical analyses, confirm that the following items are present in the figure legend, table legend, main text, or Methods section.

n/a	Confirmed	
☐	☒	The exact sample size (*n*) for each experimental group/condition, given as a discrete number and unit of measurement
☐	☒	A statement on whether measurements were taken from distinct samples or whether the same sample was measured repeatedly
☐	☒	The statistical test(s) used AND whether they are one- or two-sided<br>*Only common tests should be described solely by name; describe more complex techniques in the Methods section.*
☐	☒	A description of all covariates tested
☐	☒	A description of any assumptions or corrections, such as tests of normality and adjustment for multiple comparisons
☐	☒	A full description of the statistical parameters including central tendency (e.g. means) or other basic estimates (e.g. regression coefficient) AND variation (e.g. standard deviation) or associated estimates of uncertainty (e.g. confidence intervals)
☐	☒	For null hypothesis testing, the test statistic (e.g. *F*, *t*, *r*) with confidence intervals, effect sizes, degrees of freedom and *P* value noted<br>*Give P values as exact values whenever suitable.*
☒	☐	For Bayesian analysis, information on the choice of priors and Markov chain Monte Carlo settings
☒	☐	For hierarchical and complex designs, identification of the appropriate level for tests and full reporting of outcomes
☐	☒	Estimates of effect sizes (e.g. Cohen's *d*, Pearson's *r*), indicating how they were calculated

Our web collection on statistics for biologists contains articles on many of the points above.

Software and code

Policy information about availability of computer code

Data collection	TCGA-PAAD (TCGAbiolinks), Gepia2, Enrichr
Data analysis	Seurat (v3.0.1), scVelo, Gepia2, Kaplan-Meier Plotter, Enrichr, IMARIS 9.3 software, Graph Prism (v7.0.4), ImageJ (v1.53s), Flowjo (v10).<br>Customized R codes used for scRNA-seq: DOI - 10.5281/zendo.7104075.

For manuscripts utilizing custom algorithms or software that are central to the research but not yet described in published literature, software must be made available to editors and reviewers. We strongly encourage code deposition in a community repository (e.g. GitHub). See the Nature Portfolio guidelines for submitting code & software for further information.

Data

Policy information about availability of data

All manuscripts must include a data availability statement. This statement should provide the following information, where applicable:

- Accession codes, unique identifiers, or web links for publicly available datasets
- A description of any restrictions on data availability
- For clinical datasets or third party data, please ensure that the statement adheres to our policy

The raw files of single-cell RNA sequencing data in this study have been deposited in the Gene Expression Omnibus database under accession code GSE189753 [https://www.ncbi.nlm.nih.gov/geo/query/acc.cgi?acc=GSE189753].

Human research participants

Policy information about studies involving human research participants and Sex and Gender in Research.

Reporting on sex and gender	No sex and gender analyses were performed.
Population characteristics	13 male patients and 7 female patients with different stages of PDAC were enrolled for this study. All the patients are Asian. Supplementary Table 2 includes further information of these PDAC patients.
Recruitment	Patients diagnosed with PDAC, based on the final results of the pathology report, were included in the study. Patients who were treated with neoadjuvant chemotherapy or radiation therapy were excluded. Once patients meet these conditions, patients were randomly recruited under authorization no. SNUH 1705-031-852. There is no selection bias in the recruitment.
Ethics oversight	This study protocols were approved by Institutional Review Board (IRB) of Seoul National University Hospital (Authorization no. SNUH 1705-031-852) for human studies. For human studies, 20 of patients diagnosed with PDAC were included, and informed consent was obtained by all the participants.

Note that full information on the approval of the study protocol must also be provided in the manuscript.

Field-specific reporting

Please select the one below that is the best fit for your research. If you are not sure, read the appropriate sections before making your selection.

☒ Life sciences ☐ Behavioural & social sciences ☐ Ecological, evolutionary & environmental sciences

For a reference copy of the document with all sections, see nature.com/documents/nr-reporting-summary-flat.pdf

Life sciences study design

All studies must disclose on these points even when the disclosure is negative.

Sample size	For animal assay, sample sizes were selected empirically from previous experimental experience with similar assays, and/or from sizes generally employed in the field.<br>For cell assay, sizes were selected empirically from previous experimental experience with similar assays.
Data exclusions	No data was excluded.
Replication	Data are representative of at least 3 independent experiments, and all attempts at replication were successful.
Randomization	For animal assay, seven to ten week-old C57BL/6N male mice were randomly assigned to different treatment groups.
Blinding	The investigators were blinded to group allocation during data collection and/or analysis.

Reporting for specific materials, systems and methods

We require information from authors about some types of materials, experimental systems and methods used in many studies. Here, indicate whether each material, system or method listed is relevant to your study. If you are not sure if a list item applies to your research, read the appropriate section before selecting a response.

Materials & experimental systems

n/a	Involved in the study	
☐	☒	Antibodies
☐	☒	Eukaryotic cell lines
☒	☐	Palaeontology and archaeology
☐	☒	Animals and other organisms
☒	☐	Clinical data
☒	☐	Dual use research of concern

Methods

n/a	Involved in the study	
☒	☐	ChIP-seq
☐	☒	Flow cytometry
☒	☐	MRI-based neuroimaging

Antibodies

Antibodies used	Fluorescence-conjugated primary antibodies used for FACS (1:200 dilution) :<br>CD274 (BD, cat#745616, Clone: MIH5, BUV395), CD3 (Biolegend, cat#740268, Clone: 17A2, BUV395), CD19 (Biolegend, cat#115534,

Clone: 6D5, PerCP-Cy5.5), B220 (Biolegend, cat#103224, Clone: RA3-6B2, APC-Cy7), NK1.1 (Biolegend, cat#108732, Clone: PK136, BV-421), CD45 (eBiosciences, cat#45-0451-82, Clone: 30-F11, PerCP-Cy5.5), CD86 (Biolegend, cat#105036, Clone: GL-1, PE-TR), CD4 (Biolegend, cat#100559, Clone: RM4-5, BV510), CD25 (Biolegend, cat#102049, Clone: PC61, BV711), CD49b (Biolegend, cat#108906, Clone: DX5, FITC), CD8a (Biolegend, cat#100708, Clone: 53-6.7, PE), Foxp3 (eBiosciences, cat#25-5773-82, Clone: FJK-16S, PE-Cy7), PD-1 (eBioscience, cat#17-9985-82, Clone: J43, APC), I-A/I-E (Biolegend, cat#107622, Clone: M5/114.15.2, Alexa 700), Epcam (Biolegend, cat#118225, Clone: G8.8, BV421), CD140a (Biolegend, cat#135906, Clone: APA5, PE), CD304 (Biolegend, cat#145218, Clone: 3E12, PE-TR), TNF-alpha (Biolegend, cat#506349, Clone: MP6-XT22, BV711), IL-2 (Biolegend, cat#503822, Clone: JES6-5H4,PerCP-Cy5.5), Granzyme B (eBioscience, cat#61-8898-82, Clone: NGZB, PE-TR), IL-4 (Biolegend, cat#504118, Clone: 11B11, PE-Cy7), Perforin (Biolegend, cat#154304, Clone: S16009A, APC), IL-17A (Biolegend, cat#506914, Clone: TC11-18H10.1, Alexa700), IFN-gamma (Biolegend, cat#505850, Clone: XMG1.2, APC-Cy7), alphaSMA (Novus Biologicals, cat#NBP2-34522AF647, Clone: 1A4/asm-1, Alexa Fluor 647), CD141 (eBioscience, cat#25-1411-82, Clone : LS17-9, PE-Cy7). Phospho-SMAD2 (Bioss, Rabbit polyclonal, cat#bs-3420R). Phospho-SMAD3 (Bioss, Rabbit polyclonal, cat#bs-3425R), Phospho-STAT3 (Biolegend, cat#651010, Clone: 13A3-1, BV421).
Primary antibodies used for IHC:
anti-a-SMA (Polyclonal, Novus Biologicals, cat#NB300-978, 1:200), anti-NRP1 (EPR3113 clone, Abcam, cat#ab81321, 1:200), anti-VEGFR1 (Y103, Abcam, cat#ab32152, 1:250), anti-VEGFR2 (B309.4, Invitrogen, cat# MA5-15157, 1:4000), anti-PD-L1 (E1L3N, Cell Signaling, cat#13684, 1:200), anti-VEGFA (VG-1, Abcam, cat#ab1316, 5ug/ml), anti-PlGF (polyclonal, Abcam, cat#ab196666, 1:50).
Primary antibodies used for IF (1:200 dilution):
anti-CD31 (2H8, Merck, cat#MAB1398Z, 1:200), anti-PDGFR-β (APB5, Invitrogen, cat#14-1402-82, 1:200), and anti-NG2 (Polyclonal, Merck, cat#AB5320, 1:200).
Secondary antibodies used for IF:
TRITC-conjugated secondary antibody (Invitrogen, cat#A18894, 1:1000), AlexaFluor488- conjugated secondary antibody (Invitrogen, cat#A-11008, 4ug/ml), and AlexaFluor647-conjugated secondary antibody (Invitrogen, cat#A-21451, 2ug/ml).

Validation

Signals of all the fluorescence-conjugated antibodies were validated via fluorescence-minus-one (FMO) controls and/or IgG controls with mouse tumor and spleen tissues.

Eukaryotic cell lines

Policy information about cell lines and Sex and Gender in Research

Cell line source(s)

Expi293F cell line was purchased from ThermoFisher (Thermo, A14527).
Pan02 cell line was obtained from National Cancer Institute (NCI, #0509770).
Murine PDAC KPC001 cells (KrasG12D p53R172H/+) were kindly provided by Dr. Yves Boucher (Massachusetts General Hospital, Boston). The primary KPC cell line, KPC001 was isolated from the tumor of a 5- to 6-month-old genetically engineered mouse model of KRASG12DP53R172HPdx-1-Cre (KPC) mice (Sharma et al., 2020, J Clin Invest).

Authentication

None of the cell lines were manually authenticated.

Mycoplasma contamination

All cells used in this study were tested for mycoplasma contamination.

Commonly misidentified lines
(See ICLAC register)

No commonly misidentified line was used in this study.

Animals and other research organisms

Policy information about studies involving animals; ARRIVE guidelines recommended for reporting animal research, and Sex and Gender in Research

Laboratory animals

Experiments were performed on C57BL/6N mice, 7-10 weeks, and mice were kept under specific pathogen-free conditions with free access to food and water and mice were maintained in 12 hour light/dark cycle with 23 degree celsius ambient temperature and 40% humidity.

Wild animals

This study did not involve wild animals.

Reporting on sex

No sex analysis was performed.

Field-collected samples

All mice were housed in a ventillated temperature-controlled environment (median temperature 23 °C, humidity 65%), under a 12-hour light/dark cycle, with a free access to food and water. Mouse cages were changed weekly. No animals were excluded.

Ethics oversight

All experimental protocols were approved by the Institutional Animal Care and Use Committee (IACUC) of Seoul National University (authorisation no. SNU-200601-1-3).

Note that full information on the approval of the study protocol must also be provided in the manuscript.

Flow Cytometry

Plots

Confirm that:

- [x] The axis labels state the marker and fluorochrome used (e.g. CD4-FITC).
- [x] The axis scales are clearly visible. Include numbers along axes only for bottom left plot of group (a 'group' is an analysis of identical markers).
- [x] All plots are contour plots with outliers or pseudocolor plots.
- [x] A numerical value for number of cells or percentage (with statistics) is provided.

Methodology

Sample preparation	1. The tumour tissues were resected, chopped into small pieces, and digested in a 5% CO2 incubator at 37 °C for 20 min with fresh RPMI-1640 (Biowest, L0498) containing collagenase type IV (Worthington Biochemical, LS004189, 1 mg/mL), hyaluronidase (Sigma, H6254, 1 mg/mL), and DNase I (Sigma, DN25, 1.5 mg/mL). The digested tissues were minced, filtered through a 70 µm cell strainer, and made into single-cell suspensions.<br>2. Mouse blood was obtained by cardiac puncture using 29 guage syringe and stored in pre-EDTA coated bottle. Subsequently, RBC lysis was performed twice with 10-fold ACK buffer.<br>3. Mouse spleen was minced and filtered through a 70 µm cell strainer, and made into single-cell suspensions.<br>4. For isolation of bone marrow cells, hind limb of mouse was removed with removing muscle and fibrous tissues. Cut both femur ends with scissors and flush out the marrows into the cell culture dish of RPMI with 10% FBS.
Instrument	BD FACSymphony and BD LSRFortessa were used for data collection.
Software	Flowjo V10
Cell population abundance	The single-cell suspensions (2 × 10^6 cells) from the tumours, blood, spleen, and bone marrow were used.
Gating strategy	Supplementary Figure 1:<br>PDGFRA+ CAF was defined as FSC-SSC subset/single cell/single cell/live cell/CD45- non-immune cell/Epcam- CD31- cell/ PDGFRA+ cell<br>Supplementary Figure 2:<br>Cancer cell was defined as FSC-SSC subset/single cell/single cell/live cell/CD45- non-immune cell/Epcam+ CD31- cell<br>Endothelial cell was defined as FSC-SSC subset/single cell/single cell/live cell/CD45- non-immune cell/Epcam- CD31+ cell<br>Lymphoid cell was defined as FSC-SSC subset/single cell/single cell/live cell/CD45+ cell/CD11b- cell<br>Myeloid cell was defined as FSC-SSC subset/single cell/single cell/live cell/CD45+ cell/CD11b+ cell<br>Supplementary Figure 4:<br>PDGFRA+ CAF was defined as FSC-SSC subset/single cell/single cell/live cell/CD45- non-immune cell/Luciferase- CD31- cell/ PDGFRA+ cell<br>CD4 T cell was defined as FSC-SSC subset/single cell/single cell/live cell/CD45+ cell/CD3+ cell/CD4+ cell<br>CD8 T cell was defined as FSC-SSC subset/single cell/single cell/live cell/CD45+ cell/CD3+ cell/CD8+ cell<br>Treg was defined as FSC-SSC subset/single cell/single cell/live cell/CD45+ cell/CD3+ cell/CD4+ cell/Foxp3+ CD25+ cell<br>B cell was defined as FSC-SSC subset/single cell/single cell/live cell/CD45+ cell/CD3- cell/B220+ cell<br>NK cell was defined as FSC-SSC subset/single cell/single cell/live cell/CD45+ cell/CD3- cell/B220- cell/CD49b+ NK1.1+ cell<br>Supplementary Figure 9:<br>CAF-2 was defined as FSC-SSC subset/single cell/single cell/live cell/CD45- non-immune cell/Epcam- CD31- cell/PDPN+ cell/ CD141+ MHCII- cell

- [x] Tick this box to confirm that a figure exemplifying the gating strategy is provided in the Supplementary Information.
